# Are you for real, *Tsukamurella*? Novel *Tsukamurella* species isolated from a patient with primary myelofibrosis

**DOI:** 10.1128/asmcr.00224-25

**Published:** 2026-03-19

**Authors:** James Lee, Matthew D. Surette, Mary Czech, Adrian M. Zelazny

**Affiliations:** 1 Department of Laboratory Medicine, National Institutes of Health (NIH) 2511, Bethesda, Maryland, USA; 2 National Institute of Allergy and Infectious Diseases, National Institutes of Health (NIH) 2511, Bethesda, Maryland, USA; Rush University Medical Center, Chicago, Illinois, USA

**Keywords:** genotypic identification, MALDI-TOF MS, targeted sequencing, whole-genome sequencing, *Tsukamurella*

## Abstract

**Background:**

*Tsukamurella* species, often considered colonizers or contaminants, are increasingly recognized as opportunistic pathogens associated with catheter-related bloodstream infections, pneumonia, meningitis, and keratitis. Diagnosis of *Tsukamurella* pulmonary infections can be challenging. There are no established clinical guidelines due to the rarity of such infections, and accurate species-level identification of *Tsukamurella* remains difficult due to high interspecies similarities.

**Case Summary:**

A 64-year-old man with primary myelofibrosis presented for infectious disease evaluation prior to hematopoietic cell transplant (HCT). Although he had past tuberculosis (TB) exposure, he was asymptomatic, and chest CT was normal except for two stable left lower-lobe subpleural nodules noted earlier. Multiple respiratory cultures grew a modified acid-fast positive rod identified by MALDI-TOF MS and targeted sequencing as *Tsukamurella* spp., but further speciation was not possible, prompting whole-genome sequencing (WGS). Comparative genomic analysis demonstrated that the isolate represented a novel *Tsukamurella* species, with 91.32% average nucleotide identity to its nearest relative, *Tsukamurella sputi*. Given the absence of clinical or radiographic disease, the findings were most consistent with colonization, and no treatment was initiated. Twenty-three days after culture collection, the patient underwent a reduced-intensity mismatched unrelated HCT without complications. Post-transplant, he showed no evidence of *Tsukamurella* infection.

**Conclusion:**

Despite meeting microbiologic criteria for non-tuberculous mycobacteria pulmonary disease, this case underscores that clinical and radiographic context is essential when interpreting *Tsukamurella* isolated from respiratory sources. Accurate species identification requires a tiered approach including MALDI-TOF MS and targeted sequencing. Determination of novel species requires WGS and appropriate bioinformatic analysis.

## INTRODUCTION

*Tsukamurella* species are gram-positive, partially acid-fast, aerobic bacilli in the order Actinomycetales. *Tsukamurella* can be isolated from diverse environmental sources, including soil, water, sludge, and wastewater. Clinically, *Tsukamurella* spp. are rare opportunistic pathogens, most often associated with catheter-related bloodstream infection and pneumonia (1). *Tsukamurella* typically causes pulmonary infections in the elderly, the immunocompromised, and those with chronic lung disease, with symptoms and radiographic findings resembling mycobacterial pulmonary disease. Recent cases of respiratory infections from diverse *Tsukamurella* species (eg., *T. pulmonis*, *T. sputi*, *T. tyrosinosolvens*, and *T. inchonensis*) highlight its emergence as an opportunistic pathogen requiring species-level identification (2–6).

Guidelines for the diagnosis of *Tsukamurella* pulmonary infections and species identification are limited. Because aerobic actinomycetes are infrequently pathogenic and their isolation from respiratory samples does not always correlate with clinical disease, CLSI recommends species identification only for clinically significant infections (7). Accurate identification of *Tsukamurella* remains challenging due to their limited phenotypic reactivity. Targeted gene sequencing of 16S rRNA and other genes (*groEL*, *rpoB*, *secA1*, and *ssrA*) improves species discrimination but is hindered by high interspecies similarity. MALDI-TOF MS can identify some species, although reliable use often requires supplementation of commercial databases with spectra from reference strains (8).

In this study, we report the isolation of a novel *Tsukamurella* species from multiple respiratory cultures obtained from a 64-year-old patient with primary myelofibrosis. Identification was achieved through bioinformatic analysis of whole-genome sequencing (WGS) data. We discuss the challenges of species-level identification of *Tsukamurella* spp. using MALDI-TOF MS and targeted sequencing. Finally, we outline experimental and bioinformatic approaches for identifying novel bacterial species in the clinical microbiology laboratory.

## CASE PRESENTATION

The patient was a 64-year-old male with primary myelofibrosis who presented to the Infectious Diseases Clinic for screening pre-hematopoietic cell transplant (HCT). He had no respiratory or systemic symptoms. He was diagnosed with primary myelofibrosis 14 months before presentation and had received ruxolitinib for 6 months and momelotinib for 8 months.

**Fig 1:**
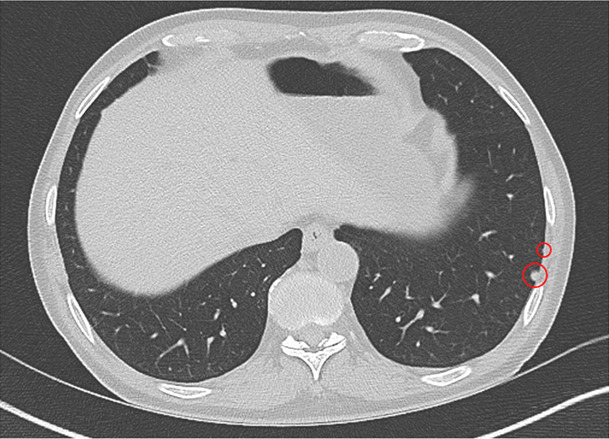
CT chest with long-term and stable subcentimeter pulmonary nodules.

He was originally from rural Ethiopia, had a remote exposure to a family member with active tuberculosis (TB), and immigrated to the United States 12 months prior to presentation. The physical examination was unremarkable. He was anemic, but white blood cell, neutrophil, and lymphocyte counts were normal. Both QuantiFERON-TB Gold Plus and T-SPOT TB were negative. CT chest imaging showed two stable left lower lobe subpleural nodules (0.5 and 0.2 cm, Fig. 1) unchanged from prior scans 8 years earlier, although the oldest images were unavailable for review.

Given the presence of these radiographic abnormalities and reported TB exposure, further diagnostic evaluation for tuberculosis was performed. Three induced sputa samples were collected over the course of 3 days and processed for AFB smears and cultures. Samples were inoculated into MGIT tubes (Remel; Lenexa, KS) containing Middlebrook 7H9, OADC, and PANTA growth supplements and Middlebrook 7H11 agar yielding modified acid-fast rods (less than 20 colonies) in all cultures. Time to positivity of MGIT was 9 days. Two sputa were tested for *Mycobacterium tuberculosis* using the Xpert MTB/RIF molecular assay, and both were negative.

Identification of the isolate was attempted via Bruker MTB Smart MALDI-TOF MS (Billerica, MA), yielding low-confidence scores for several *Tsukamurella* species, including *T. paurometabola* (1.76) and *T. pulmonis* (1.55). The isolate was reported as a “presumptive *Tsukamurella* sp.” Given the planned intensification of immunosuppression following HCT, the isolate was referred for targeted Sanger sequencing of the 16S rRNA, *rpoB*, and *secA* genes for genus- and species-level identification (Table 1). The organism showed nearly 100% identity to *T. pulmonis*, *T. tyrosinosolvens*, and *T. paurometabola* by the 16S rRNA gene. In contrast, *sec*A and *rpo*B sequences diverged, with closest matches to *T. pulmonis* at 97.92% and 94.68% identity, respectively, suggesting the isolate was genetically distinct from other *Tsukamurella* species.

**TABLE 1:** Targeted Sanger sequencing results for *rpoB*, *secA*, and 16S rDNA^*a*^

Fragment	Organism name	NCBI accession no.	% Identity (match/total)
*rpoB*	*Tsukamurella pulmonis* TP-B0596	AP025457	97.92 (471/481)
	*Tsukamurella tyrosinosolvens* NCTC 13231 T	LR134465	96.27 (464/481)
	*Tsukamurella paurometabola* NCTC 10741 T	LR131273	96.05 (462/481)
*secA*	*Tsukamurella pulmonis* 03-028-3227 (partial)	GU179144.1	94.68 (445/470)
	*Tsukamurella paurometabola* NCTC10741	LR131273.1	92.79 (476/513)
	*Tsukamurella tyrosinosolvens* QAP 01-332-3148 (partial)	GU179146.1	92.77 (436/470)
16S rDNA	*Tsukamurella hominis* HKU65	NR_159884.1	99.85 (648/649)
	*Tsukamurella ocularis* HKU63	NR_159883.1	99.85 (648/649)
	*Tsukamurella pulmonis* IMMIB D-1321 (partial)	NR_029302.1	99.84 (626/627)

^*a*^ Fragments of the *rpoB *(481 bases), *secA *(519 bases), and 16S rDNA (649 bases) were amplified and sequenced from the clinical isolate gDNA. The sequenced reads were analyzed by NCBI BLAST for identification.

**Fig 2:**
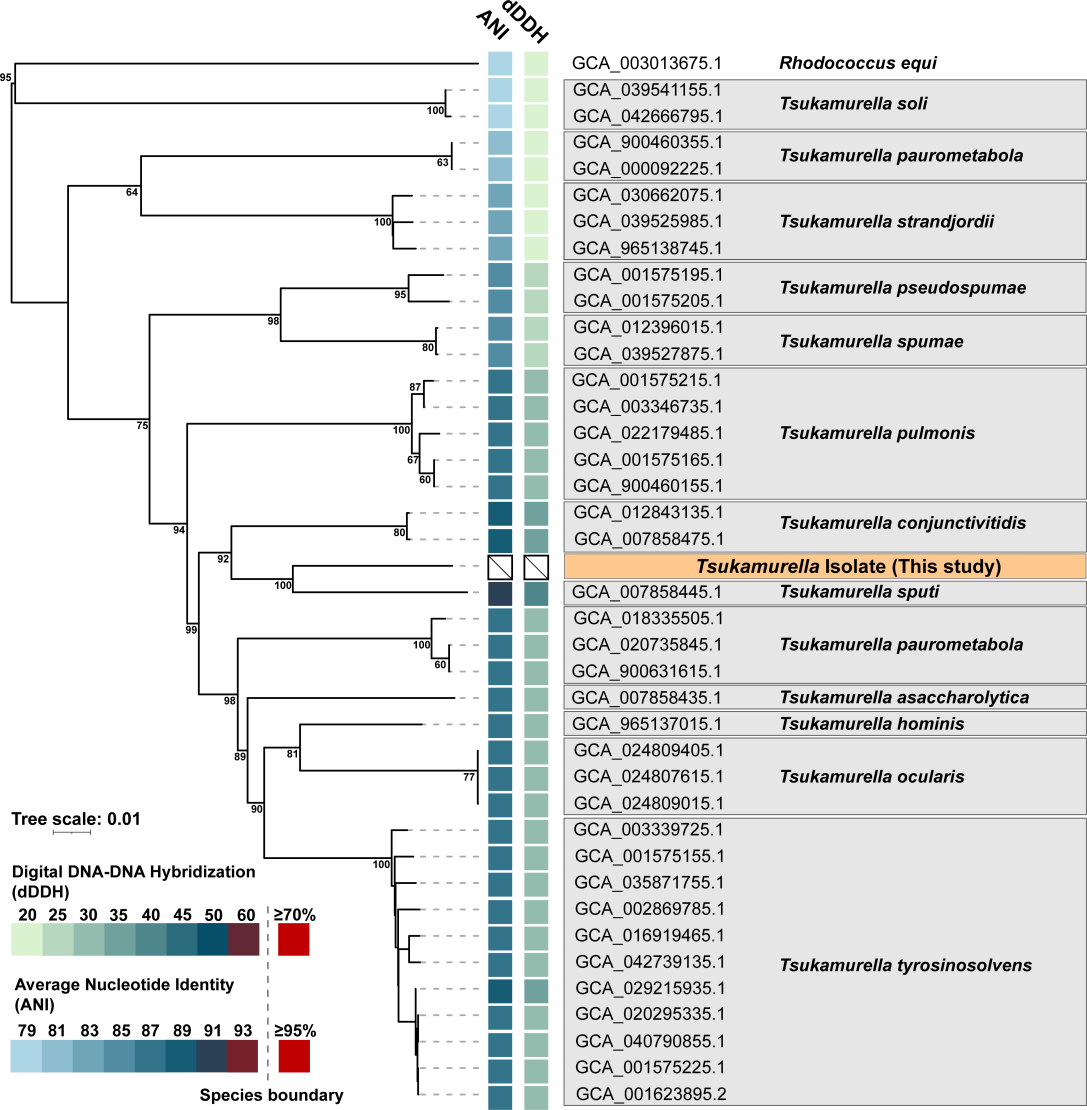
Phylogenetic analyses of the novel *Tsukamurella* species and related taxa. A phylogenetic tree was constructed from reference *Tsukamurella* genomes using the TYGS webserver visualized using the interactive tree of life (12). The ANI and dDDH value for each genome relative to our isolate are displayed at the tips of the tree. No *Tsukamurella* species is close to the widely used species boundaries of either metric (dDDH ≥70%, ANI ≥95%), indicating our isolate belongs to a novel *Tsukamurella* species. Branch lengths are scaled based on the Genome BLAST distance phylogeny method, and the numbers above branches correspond to pseudo-bootstrap support values (11).

To fully characterize the isolate, WGS was performed. Genomic DNA was extracted using the QIAGEN DNeasy UltraClean Microbial Kit, and sequencing libraries were prepared and run on an in-house GridIon sequencer. To increase depth, genomic DNA was also sequenced commercially (Psomagen) on a Nanopore platform. *De novo* assembly with Flye generated a complete 4.7 Mb circular genome with >500× coverage. Whole-genome phylogenetic analysis using the Type (Strain) Genome Server (TYGS) and the Genome Taxonomy Database Toolkit (GTDB-tk) both classified the isolate as a novel species (Fig. 2). Among *Tsukamurella* species, *T. sputi* was the most closely related to this isolate, with an average nucleotide identity (ANI) of 91.32% (GTDB-tk) and a digital DNA–DNA hybridization (dDDH) of 44% (9–11).

One culture also showed concurrent growth of *M. gordonae/paragordonae*, which was considered a contaminant. Antimicrobial susceptibility testing was performed using the Sensititre Nocardia Susceptibility Testing Plate and interpreted according to CLSI M24S guidelines (7). The isolate was susceptible to amikacin, ciprofloxacin, clarithromycin, imipenem, moxifloxacin, and trimethoprim/sulfamethoxazole (Table 2).

**TABLE 2:** Antibacterial susceptibility testing results of the novel *Tsukamurella* sp.

Antibiotic	MIC^*a*^ (µg/mL)	Interpretation^*b*^
Amikacin	≤0.5	S
Ciprofloxacin	0.25	S
Clarithromycin	0.12	S
Imipenem	0.25	S
Moxifloxacin	0.12	S
Trimethoprim-sulfamethoxazole	≤0.06/1.19	S

^*a*^ MIC, minimal inhibitory concentration.

^*b*^ Tentative breakpoints for aerobic actinomycetes, derived from *Nocardia* spp. (CLSI M24S).

*Tsukamurella* isolated from respiratory samples was interpreted as colonization rather than infection, given the absence of pneumonia-related clinical symptoms and radiographic infiltrates (13–15). Twenty-three days following collection of the respiratory cultures, the patient underwent successful reduced-intensity mismatched unrelated HCT. No clinical symptoms or radiographic findings consistent with *Tsukamurella* infection were observed pre- or post-HCT, and no antibacterial therapy with activity against *Tsukamurella* was administered within 30 days post-HCT (approximately 2 months after the initial positive respiratory culture).

## DISCUSSION

Presence of *Tsukamurella* in non-sterile sites often represents colonization or contamination, but likelihood of clinical significance increases with certain host risk factors (immunocompromise, lung conditions, trauma, or surgery). Species such as *T. pulmonis*, *T. tyrosinosolvens*, and *T. paurometabola* have caused pneumonia in immunocompromised patients (post-transplant, cancer, and HIV) (16). Cases vary in presentation from right upper lobe/bilateral community-acquired pneumonia (CAP) and bacteremic pneumonia to cavitary disease and TB-like syndromes. *Tsukamurella* pneumonia may be underdiagnosed, particularly in TB-endemic countries, due to its clinical presentation, which resembles tuberculosis.

The identification of *Tsukamurella* to species level can be challenging for clinical microbiology laboratories. Biochemical tests have limited usefulness for identifying aerobic actinomycetes, especially at the species level (17, 18). MALDI-TOF MS is increasingly used for identification; however, it is typically sufficient only for genus-level identification of *Tsukamurella*. Species-level identification by MALDI-TOF MS is prone to errors but can be improved upon supplementation of databases with spectra from reference strains (2, 8). The best match to our isolate was *T. paurometabola*, with a relatively low score of 1.76, which provides genus-level identification (>1.7) but not speciation.

The 16S rRNA gene is widely used for bacterial identification, but additional targets such as *rpo*B, *secA*, *ssrA*, and *groEL* provide higher discrimination for *Tsukamurella* speciation (2). CLSI MM18 guidelines for identification of aerobic actinomycetes recommend 99.0%–99.5% and ≥99.6% similarity by 16S rDNA for genus- and species-level identification, with >0.4% separation between species (19). CLSI MM18 guidelines lack cutoffs for other genes, but reported thresholds include 99.3% and 98.3% for *rpo*B in slowly and rapidly growing mycobacteria and >99% for *sec*A across all mycobacterial species. Studies using 16S rDNA and *rpo*B show that rapidly growing mycobacteria with <97% similarity to their closest species may represent novel species (20, 21). Our targeted sequencing results using 16S rDNA (99.8% similarity to multiple species), *rpo*B (97.92%), and *sec*A (94.68%) showed conflicting species assignments and low overall similarity to described *Tsukamurella* species. Although organism identification as “*Tsukamurella* species” was concluded with targeted sequencing, further investigation was pursued for academic purposes and in anticipation of any change in the patient’s clinical presentation with HCT.

Whole-genome sequencing (WGS) is increasingly used in clinical microbiology for bacterial identification, genotyping, and antimicrobial resistance prediction. Species delineation has long relied on genomic relatedness, traditionally measured by DNA–DNA hybridization (DDH), with ≥70% DDH indicating the same species. As WGS has become more accessible, genome-wide comparative methods are now widely used. Average nucleotide identity (ANI), which measures shared nucleotide identity between genomes, provides far higher taxonomic resolution than single- or multi-gene sequencing. A ~95% ANI threshold generally defines species boundaries and corresponds to the traditional 70% DDH cutoff (22–26). Genome-based taxonomic placement is commonly performed using tools such as the Genome Taxonomy Database Toolkit (GTDB-tk) and Type (Strain) Genome Server (TYGS) (9–11). GTDBtk uses ANI for species-level discrimination, whereas TYGS predicts DDH values (dDDH) using an ANI-like algorithm, enabling use of the traditional 70% DDH species cutoff. For clinical microbiology laboratories, it is important to note that unlike GTDB-tk, which maintains its own taxonomy, TYGS is linked to the List of Prokaryotic Names with Standing in Nomenclature.

Isolation of environmental organisms such as *Tsukamurella* from non-sterile sites, including respiratory samples, should be interpreted in clinical context. For comparison, NTM pulmonary disease requires clinical, radiographic, and microbiologic criteria (27), as NTM can appear in respiratory specimens from contamination or colonization, and some patients with positive cultures show no progressive disease. Although ATS/IDSA NTM diagnostic criteria are not routinely applied to *Tsukamurella*, elements of this framework, including repeated recovery from non-sterile specimens and compatible clinical and radiologic findings, are often informally considered.

Diagnostic criteria for NTM pulmonary infection include isolation of the same species from at least two sputum cultures; although no criteria exist for *Tsukamurella*, three positive cultures would meet and exceed this microbiological threshold (1–11). Nevertheless, clinical findings in our patient were not consistent with pulmonary infection. Although chest CT showed nodules, these remained unchanged from prior imaging. Therefore, *Tsukamurella* was deemed a colonizer rather than a pathogen, and targeted treatment was not initiated.

Given the infrequency of infections due to *Tsukamurella*, optimal treatment regimens are not universally established. *Tsukamurella* spp. are generally susceptible to amikacin, ciprofloxacin, imipenem, doxycycline, linezolid, and sulfamethoxazole, but resistant to penicillin, piperacillin/tazobactam, and cephalosporins, with limited data suggesting higher resistance in *T. paurometabola* and *T. pulmonis* (2, 28, 29). As we gain more experience with *Tsukamurella*, species-level identification and susceptibility testing may reveal species-specific disease associations and antimicrobial profiles. Accurate identification may therefore help diagnostic challenges and guide more targeted antibiotic therapy and potentially improve treatment outcomes.

## Data Availability

The genome sequence data were deposited in NCBI under BioProject no. PRJNA1400060.
